# Recovery of the PHA Copolymer P(HB-*co*-HHx) With Non-halogenated Solvents: Influences on Molecular Weight and HHx-Content

**DOI:** 10.3389/fbioe.2020.00944

**Published:** 2020-08-07

**Authors:** Moritz Bartels, Björn Gutschmann, Torsten Widmer, Thomas Grimm, Peter Neubauer, Sebastian L. Riedel

**Affiliations:** ^1^ANiMOX GmbH, Berlin, Germany; ^2^Technische Universität Berlin, Institute of Biotechnology, Chair of Bioprocess Engineering, Berlin, Germany

**Keywords:** polyhydroxyalkanoate, recovery, non-halogenated solvents, multi-stage extraction, molecular weight, medium chain length PHA, P(HB-*co*-HHx), *Ralstonia eutropha*

## Abstract

Biodegradable and biocompatible polyhydroxyalkanoates (PHAs) are promising alternatives to conventional plastics. Based on the chain length of their monomers they are classified as *short chain length* (*scl-*) or *medium chain length* (*mcl-*) PHA polymers. The type of monomers, the composition and the molecular weight (MW) define the polymer properties. To accelerate the use of PHA as a bulk material, the downstream associated costs need to be minimized. This study focuses on the evaluation of non-halogenated solvents, especially acetone as a *scl*-PHA non-solvent, for the recovery of poly(3-hydroxybutyrate-*co*-3-hydroxyhexanoate) – P(HB-*co*-HHx) – with an *mcl*-HHx content >15 mol% and a MW average (*M*_w_) < 2 × 10^5^ Da. Solvents and precipitants were chosen regarding zeotrope formation, boiling point differences, and toxicity. Non-halogenated solvent-precipitant pairs were evaluated regarding the MW characteristics (MWCs) of the extracted polymer. Acetone and 2-propanol as a low toxic and zeotropic solvent-precipitant pair was evaluated at different extraction temperatures and multiple extraction times. The extraction process was further evaluated by using impure acetone for the extraction and implementing a multi-stage extraction process. Additionally, P(HB-*co*-HHx) extracted with three different solvents was characterized by ^1^H and ^13^C-APT NMR. The screening of precipitants resulted in a negative influence on the MWCs by ethanol precipitation for extractions with acetone and ethyl acetate, respectively. It was observed, that extractions with acetone at 70°C extracted a higher fraction of PHA from the cells compared to extractions at RT, but the *M*_w_ was decreased by 9% in average. Acetone with a 2-propanol fraction of up to 30% was still able to extract the polymer 95% as efficient as pure acetone. Additionally, when acetone and ethyl acetate were used in a multi-stage extraction process, a two-stage process was sufficient to extract 98–99% of the polymer from the cells. ^1^H and ^13^C-APT NMR analysis confirmed the monomer fraction and structure of the extracted polymers and revealed a random copolymer structure. The presented strategy can be further developed to an ecological and economically feasible PHA downstream process and thus contributes to the commercialization of low-cost PHAs.

## Introduction

Plastic pollution of land and sea is a ubiquitous problem, which consequently results in microplastic in the animal and human food chain. Biodegradable alternatives, such as microbial synthesized polyhydroxyalkanoates (PHAs), have the potential to partially substitute fossil-based and non-biodegradable plastics (Sudesh et al., 2000; Chen, 2009; Brigham and Sinskey, 2012; Kourmentza et al., 2017; Brigham, 2018). Production of PHAs requires the isolation of the intracellular polymers from other biomass components. Several extraction strategies have been employed and comprehensively reviewed in the last years: solvent extraction (halogenated, non-halogenated), chemical disruption methods (hypochlorite, alkaline, surfactants) in combination with enzymes, mechanical disruption (bead mill, high pressure homogenization, ultrasonication), supercritical fluid extraction, aqueous two-phase systems, air classification (Koller et al., 2013b; Madkour et al., 2013; Pérez-Rivero et al., 2019), and biological approaches using insects and other animals (Murugan et al., 2016; Kunasundari et al., 2017; Ong et al., 2017; Zainab-L and Sudesh, 2019). However, solvent-based extraction is still the most explored PHA recovery method and allows the recovery of high purity PHA. Large amounts of halogenated solvents – e.g. chloroform – are traditionally used in the extraction process (Pérez-Rivero et al., 2019). Especially chlorinated solvents are expensive at industrial scale and have a negative impact on the ecological footprint of the overall process. Consequently, the downstream process accounts for a large portion of the PHA production price.

Microbial synthesized PHAs can consist of various building blocks, which are classified according to their number of carbon atoms: *short-chain length* (*scl*; C ≤ 5) and *medium-chain length*

(*mcl*; 6 ≤ C ≤ 14) are mainly distinguished (Steinbüchel et al., 1992). Copolymers of *scl-* and *mcl*-building blocks, in particular poly(3-hydroxybutyrate-*co*-3-hydroxyhexanoate) – P(HB-*co*-HHx) –, show superior properties regarding the crystallinity, flexibility, ductility, toughness and processability compared to the *scl*-homopolymer polyhydroxybutyrate (PHB) (Noda et al., 2005). In addition, P(HB-*co*-HHx) exhibits short degradation times: three months in anaerobic sludge and six months under aerobic seawater conditions (Wang et al., 2018). The relevance of this copolymer gets even more apparent considering that the companies Danimer Scientific^1^ (Bainbridge, United States) and Kaneka Corporation^2^ (Osaka, Japan) recently introduced P(HB-*co*-HHx)-based products into the market.

Recent studies show that *mcl*-PHAs and their copolymers have better solubility in a range of halogen-free solvents. High yields and high product purities were achieved with ethyl acetate and acetone as extracting solvents (Jiang et al., 2006; Furrer et al., 2007; Riedel et al., 2013). Halogen-free solvents generally solve *mcl*-PHAs better than *scl*-PHAs (Terada and Marchessault, 1999), but the solubility depends on more than the side-chain length. The solubility of a polymer is a complex process, which is affected by various parameters such as temperature, pressure, and polymer concentration (Sadowski, 2004). The procedure becomes even more diverse, when a polymer that was produced by bacterial fermentation must be extracted from the biomass. Polymers often show only limited solubility in organic solvents. It is assumed that solubility is additionally affected by the molecular weight (MW) and molecular weight distribution (MWD) of the polymer, which is considered by the Hansen solubility parameters (HSPs) (Hansen, 2007). Materials with similar HSPs have high affinity for each other. It was shown that the solubility of P(HB-*co*-HHx) increases with an increasing HHx-fraction, which consequently allows PHB non-solvents to be used for P(HB-*co*-HHx) extraction (Jacquel et al., 2007).

In this study, the recovery of P(HB-*co*-HHx) from freeze-dried biomass was examined. Different non-halogenated solvents and multiple extraction conditions are compared with a focus on the MW characteristics (MWCs) of the recovered polymers.

## Materials and Methods

### Production of Cells for P(HB-*co*-HHx) Recovery

Biomass containing P(HB-*co*-HHx) was produced in a 150-L bioreactor (P150, Bioengineering AG, Wald, Switzerland) using the genetic modified bacterium *Ralstonia eutropha* Re2058/pCB113 (Budde et al., 2011b). The cells were produced from waste animal fats (ANiMOX GmbH, Berlin, Germany) as described previously (Riedel et al., 2015).

### Soxhlet Extraction General Procedure

An amount of 3 g of freeze-dried cells was extracted with 70 mL solvent (acetone and chloroform, respectively) in a Soxhlet extractor under reflux conditions for 3 h. The extract phase was recovered and concentrated by rotary evaporation. Precipitation was induced by addition of the three-fold amount of *n-*heptane. The polymer was washed with 20 mL fresh *n-*heptane and dried at 50°C for 4 h.

#### Thermal Stability Evaluation

A 40 mg sample of P(HB-*co*-HHx) was added to 2 mL chloroform in a sealed test tube and heated at 100°C in a heating block for 4 h. The MW of the heated sample was compared with the untreated sample.

### Comparison of Precipitants

An amount of 10 g of freeze-dried cells was extracted with 100 mL solvent (acetone or ethyl acetate, respectively) under reflux conditions for 3 h. The extract phase was recovered and concentrated by rotary evaporation. Precipitation was induced by addition of the three-fold amount of a PHA non-solvent (1-propanol, 2-propanol, ethanol, *n*-heptane) and incubation at 4°C for 24 h. The polymer was washed three times with 30 mL of fresh non-solvent and dried at 50°C for 4 h.

### Variation of Extraction Conditions

Polyhydroxyalkanoates was extracted from freeze-dried cells with variation of the following parameters: extraction temperature (RT or 70°C) and extraction time (0.5–6.0 h). Preliminary tests showed that a 10:1 volume to weight ratio of solvent to freeze-dried cells gives the best results for following processing steps. 10 g of freeze-dried cells and 100 mL solvent were added to a 250 mL round-bottom-flask, equipped with a reflux condenser and a magnetic stir bar. The extract was separated from cell debris by centrifugation at 3,000 × *g* for 10 min followed by filtration of the supernatant through a cellulose fiber filter (10 μm). The filtrate was concentrated by rotary evaporation until the viscosity of the solution noticeably increased. Addition of the three-fold amount of 2-propanol and incubation at 4°C for 24 h induced precipitation of the polymer. The precipitate was recovered by vacuum filtration, washed three times with 30 mL of non-solvent and dried at 50°C for 4 h.

### Extraction With Impure Solvent

1 g of freeze-dried cells was extracted with 10 mL solvent under reflux conditions for 1 h. As a solvent acetone with a defined volume fraction of 2-propanol in a range of 0–100% was used. The extract phase was recovered and precipitated by addition of 30 mL heptane and incubation at 4°C for 24 h. The recovered polymer was quantified by weighting. The extraction with pure 2-propanol was used as a reference extraction and the recovery yield was calculated relatively to the reference extraction.

### Multi-Stage Extraction

1 g of freeze-dried cells was extracted with 10 mL solvent (acetone or ethyl acetate) under reflux conditions. The extract phase was recovered and precipitated by addition of a PHA non-solvent (*n*-heptane or 2-propanol) and dried at 50°C for 4 h. The procedure was repeated two times with the respective remaining cells.

### Determination of Molecular Weight Characteristics

Molecular weight distribution was determined by size exclusion chromatography (SEC) using two sequentially coupled SEC columns (Agilent PLgel 5 μm MIXED-C 300 × 7.5 mm) and a differential refractive index detector (Merck-Hitachi, RI-Detector L-7490). Samples were prepared by dissolving 20 mg of recovered PHA in 1 mL chloroform (HPLC grade) at 40°C for 2 h. Before analysis the samples were filtered through a syringe filter (PTFE, 0.2 μm). Aliquots of 10 μL were injected and analysis was performed at ambient temperature with a flowrate of 1 mL min^–1^. Calibration was performed with five narrow polystyrene standards (Agilent Polystyrol PS-1) in the range of 30–1,037 kDa. The extracted signal data points (*n*_i_) and respective MW (*M*_i_) calculated by the calibration data were used to calculate the number average (*M*_n_), weight average (*M*_w_), and dispersity values (*Ð*) according to Eqs (1–3):

(1) $$M_{n}=\frac{\sum_{i=1}^{k}\left(n_{i}\cdot M_{i}\right)}{\sum_{i=1}^{k}n_{i}}$$

(2) $$M_{w}=\frac{\sum \left(n_{i}\cdot M_{i}^{2}\right)}{\sum \left(n_{i}\cdot M_{i}\right)}$$

(3) $$\text{–}D=\frac{M_{w}}{M_{n}}$$

### Determination of the PHA Content and Purity

The PHA content was determined using a gas chromatograph (GC) (GC-2010 Plus, Shimadzu Corp., Kyoto, Japan) equipped with a flame ionization detection (FID) and a DB-WAX column (15 m × 0.32 mm × 0.5 μm, Agilent Technologies Inc., Santa Clara, CA, United States). The samples were prepared for GC measurements by methanolysis as described previously (Budde et al., 2011a). Pure PHB and methyl-3-hydroxyhexanoate were used to yield standards in the range of 1–15 and 0.1–5 mg mL^–1^, respectively. Methyl undecanoate was used at a concentration of 1 mg mL^–1^ as an internal standard. Analytical standards were purchased at Sigma-Aldrich Corporation (St. Louis, United States). 10 μL of the sample was injected with a split ratio of 1:10 into the GC using an autosampler. The temperature of the injector and the detector were set to 200 and 180°C, respectively. A temperature gradient was used to separate the analytes: 80°C was hold for 2 min, temperature increased 7°C min^–1^ to 150°C, temperature increased 10°C min^–1^ to 220°C, and holding at 220°C for 10 min. Calibration curves of the analytical standards were used to calculate the HB and HHx contents of the samples from the respective peak areas.

### NMR of P(HB-*co*-HHx)

^1^H and ^13^C attached proton test (^13^C-APT) NMR spectra of P(HB*-co-*HHx) copolymer samples were recorded on a Bruker Avance III HD-400 spectrometer (Rheinstetten, Germany) in CDCl_3_ at RT. For the ^13^C-APT NMR experiment chemical shifts are accompanied by minus (−) (for quaternary C and CH_2_) and plus (+) (for CH and CH_3_). Integrals of the methyl group protons of HB (δ = 1.23 ppm) and HHx (δ = 0.87 ppm) were determined from the ^1^H NMR spectra to calculate the molar fraction *F* of HHx monomer according to Eq. 4:

(4) $$F_{HHx}=\frac{I_{HHx}}{I_{HHx}+I_{HB}}$$

The results of the ^13^C-APT NMR was used to calculate the randomness *D* of the polymer (Equation 5) as described by Kamiya et al. (1989):

(5) $$D=\frac{F_{HB\times HB}\cdot F_{HHx\times HHx}}{F_{HB\times HHx}\cdot F_{HHx\times HB}}$$

It allows to distinguish between block (*D* > 1), random (*D* ≈ 1) and alternating (*D* < 1) copolymers.

## Results

Polyhydroxyalkanoates polymers containing *mcl*-monomers have an increased solubility in *scl*-PHA non-solvents (Jacquel et al., 2007). In this study, non-halogenated solvents and precipitants for P(HB-*co*-HHx) recovery were evaluated according to their physical properties, toxicity and environmental risk. Non-halogenated solvent-precipitant pairs were examined regarding influences of extraction temperature and time on the MW, purity and molar HHx-content of P(HB-*co*-HHx). In addition, solvent impurities as well as a multi-stage extraction process were evaluated and the structure of the extracted P(HB-*co*-HHx) was confirmed by ^1^H and ^13^C-APT NMR.

### Solvent-Precipitant Pairs for P(HB-*co*-HHx) Recovery

Non-halogenated solvents are especially suitable for the recovery of *mcl*-PHAs (Pérez-Rivero et al., 2019). A subsequent precipitation of the solubilized PHA by a PHA non-solvent yields high purity polymer (Ramsay et al., 1994). The physical properties as well as hazard information of compounds, which can potentially be employed in the *mcl*-PHA downstream process, are shown in Table 1. An additional factor, which was considered for the solvent selection was the formation of azeotropes (Table 2). Formation of azeotropes prevents a solvent recovery by distillation and should therefore be avoided for the development of an economical process. In this study we focused therefore on the use of acetone and ethyl acetate as potential solvents for the P(HB-*co*-HHx) extraction process.

**TABLE 1:** Physical properties of compounds, which are possibly employed for PHA downstream processing.

Compound	*T*_B_ (°C)^a^	Viscosity (10^–^^3^ Pa s)^a^	Density (g cm^–^^3^)^a^	Solubility in water (g L^–^^1^)^a^	MAK (ppm)^b^	FDA class^c^	Environmental score^d^
DMK	56	0.30	0.79	Miscible	500	3	5
TCM	61	0.54	1.49	8.0	0.5	2	5
DCM	40	0.41	1.32	17.5	50	2	7
EA	77	0.43	0.89	80.0	200	3	3
MEK	80	0.40	0.80	248.0	200	3	3
MIBK	117	0.59	0.80	19.0	20	2	3
MTBE	55	0.36	0.74	51.3	50	3	5
Tol	111	0.55	0.86	0.5	50	2	3
1-PrOH	97	1.96	0.80	Miscible	200	3	n.d.
2-PrOH	83	2.04	0.78	Miscible	200	3	3
EtOH	78	1.04	0.79	Miscible	200	3	3
*n*-Hep	98	0.39	0.68	0.002	500	3	7
*n*-Hex	68	0.30	0.66	0.01	50	2	7
MeOH	65	0.54	0.79	Miscible	100	2	5

*n.d., no data available. DMK, acetone; TCM, chloroform; DCM, dichloromethane; EA, ethyl acetate; MEK, Methyl ethyl ketone; MIBK, methyl isobutyl ketone; MTBE, methyl tert-butyl ether; Tol, Toluene; 1-PrOH, 1-propanol; 2-PrOH, 2-propanol; EtOH, ethanol; n-Hep, n-heptane; n-Hex, n-hexane; MeOH, methanol. ^a^Physical property data at 25°C and 1 atm from Yaws (1999). ^b^MAK is the permissible exposure limit defined by the German research association (DFG): MAK- und BAT-Werte-Liste 2019. DFG, ISBN:978-3-527-34742-1. ^c^FDA guidance for industry Q3C August 2018, where 1 is most toxic and 3 least toxic (http://www.fda.gov/downloads/Drugs/GuidanceComplianceRegulatoryInformation/Guidances/ucm073395.pdf). ^d^Environmental scores from Prat et al. (2016), where 1 represents the least hazardous and 10 the most hazardous score.*

**TABLE 2:** Boiling point differences and azeotropic boiling points of solvent-precipitant pairs for PHA downstream processing.

Solvent	Precipitant											
	Δ*T*_B_ (K)^a^						*T*_B,Az_ (°C) (*y*_1,Az_)^b^					
	1-PrOH	2-PrOH	EtOH	*n*-Hep	*n*-Hex	MeOH	1-PrOH	2-PrOH	EtOH	*n*-Hep	*n*-Hex	MeOH
DMK	41	27	22	42	12	9	Z	Z	Z	56 (0.94)	50 (0.65)	55 (0.76)
TCM	36	22	17	37	7	4	Z	61 (0.95)	59 (0.84)	Z	60 (0.78)	55 (0.65)
DCM	57	43	38	58	28	25	Z	Z	39 (0.96)	Z	Z	38 (0.83)
EA	20	6	1	21	9	12	Z	76 (0.67)	72 (0.54)	77 (0.95)	65 (0.34)	63 (0.28)
MEK	17	3	2	18	12	15	Z	78 (0.62)	75 (0.53)	77 (0.74)	63 (0.66)	64 (0.88)
MIBK	20	34	39	19	49	52	Z	77 (0.62)	74 (0.49)	77 (0.77)	64 (0.33)	50 (0.20)
MTBE	42	28	23	43	13	10	Z	Z	54 (0.84)	Z	Z	50 (0.65)
Tol	14	28	33	13	43	46	93 (0.63)	82 (0.84)	77 (0.81)	Z	Z	64 (0.88)

*DMK, acetone; TCM, chloroform; DCM, dichloromethane; EA, ethyl acetate; MEK, Methyl ethyl ketone; MIBK, methyl isobutyl ketone; MTBE, methyl tert-butyl ether; Tol, Toluene. 1-PrOH, 1-propanol; 2-PrOH, 2-propanol; EtOH, ethanol; n-Hep, n-heptane; n-Hex, n-hexane; MeOH, methanol. Z, zeotropic mixture. ^a^Physical property data at 25°C and 1 atm from Yaws (1999). ^b^Data from Horsley (1973) and Haynes (2015).*

### Reference Extractions: Chloroform Extraction

**FIGURE 1:**
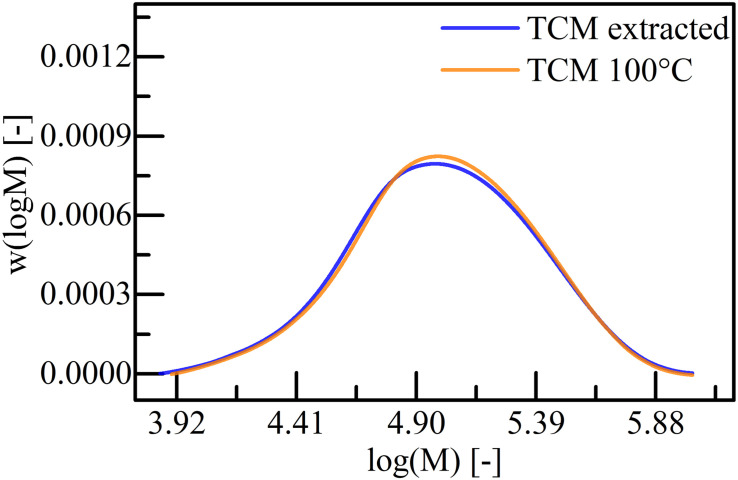
Distribution of the logarithmic molecular weight for P(HB-*co*-HHx) extracted with chloroform (TCM) at RT and incubated for 4 h at 100°C and precipitated with *n*-heptane. The extraction was performed in a Soxhlet apparatus under reflux conditions.

The length of polymer chains and their distribution are key parameters for further processing. In order to evaluate suitable extraction conditions, P(HB-*co*-HHx) produced from waste animal fats was extracted from freeze-dried cells with chloroform at RT (approximately 21°C) and the thermal stability was evaluated for 4 h at 100°C. The recovered and heated polymers were evaluated with SEC: the distributions of the logarithmic MW are displayed in Figure 1 and the results of the evaluation is shown in Table 3. The experiments yielded in a very similar MWD with an *M*_w_ of 1.41 × 10^5^ and 1.42 × 10^5^ Da, *M*_n_ of 0.70 × 10^5^ and 0.73 × 10^5^ Da, and a *Ð* of 1.9 and 2.0, for the extraction at RT and 100°C treatment respectively. Thus, a wide range of temperatures are suitable for the processing of P(HB-*co*-HHx) without affecting the MWCs, which allows a diverse downstream process and product processing development.

**TABLE 3:** Molecular weight characteristics of P(HB-*co*-HHx) recovered with chloroform from freeze-dried cells at RT (21°C) and P(HB-*co*-HHx) treated at 100°C for 4 h, respectively.

Condition	*M*_w_ (10^5^ Da)	*M*_n_ (10^5^ Da)	*Ð* (−)
Extracted (RT)	1.41	0.70	2.0
Incubated (100°C)	1.42	0.73	1.9

*Extractions were performed in a Soxhlet apparatus under reflux conditions and n-heptane was used as precipitant.*

### Evaluation of Precipitants for PHA Recovery With Non-halogenated Solvents

**FIGURE 2:**
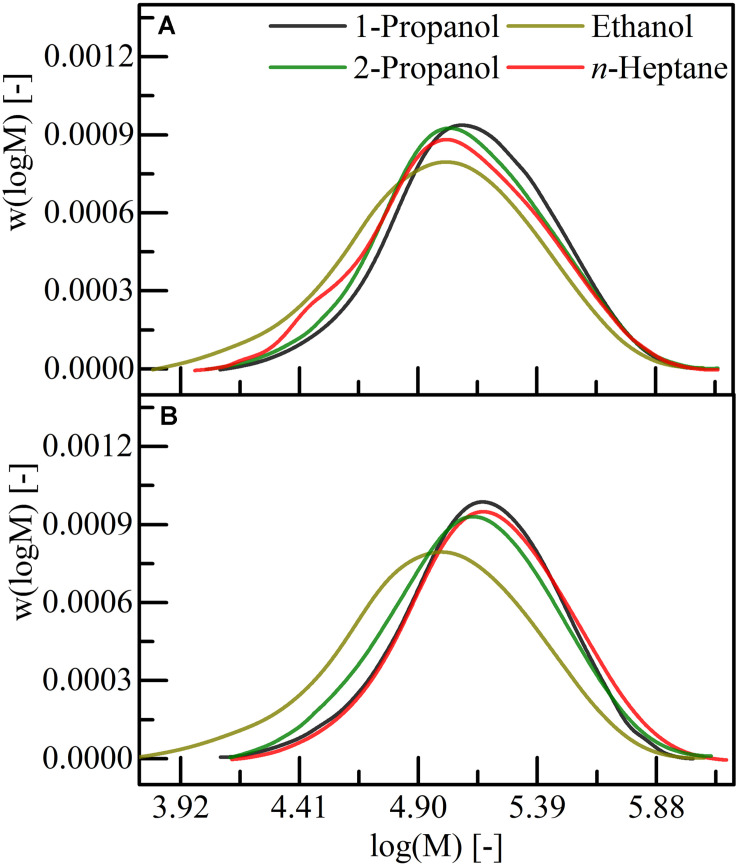
Distribution of the logarithmic molecular weight for P(HB-*co*-HHx) extracted with **(A)** acetone and **(B)** ethyl acetate. Extractions were performed under reflux conditions. 1-propanol, 2-propanol, ethanol, and *n*-heptane were used as precipitants, respectively.

With the objective to compare the MWCs of P(HB-*co*-HHx) extracted with ethyl acetate or acetone and precipitated by different solvents (1-propanol, 2-propanol, ethanol, *n*-heptane), material was extracted from freeze-dried cells. A cell solvent suspension with a cell concentration of 0.1 g mL^–1^ was used for the recovery experiments and the PHA was precipitated by adding the three-fold volume of the respective non-solvent. The distributions of the logarithmic MW are displayed in Figure 2 and the results of the evaluation is shown in Table 4. Precipitation with ethanol yielded in a broader MWD and significant lower MW for both solvents compared to precipitation with the other three non-solvent. By extraction with ethyl acetate, the recovered polymer was of slightly higher MW compared to the acetone extractions, for all other three precipitants (Table 4). Nevertheless, extraction with both solvents and precipitation with 1-propanol, 2-propanol or *n*-heptane resulted in a narrow MWD (*Ð* = 1.7–1.9) and also in a higher MW as from the chloroform/*n*-heptane recovery (Tables 3, 4). Consequently, acetone is as suitable as ethyl acetate for the recovery of P(HB-*co*-HHx) but precipitation with ethanol should be circumvented due to a drastic decrease of the MW and broader MWD.

**TABLE 4:** Comparison of molecular weight characteristics of P(HB-*co*-HHx) recovered with ethyl acetate and acetone from freeze-dried cells.

	Solvent					
	Acetone			Ethyl acetate		
Precipitant	*M*_w_ (10^5^ Da)	*M*_n_ (10^5^ Da)	*Ð* (−)	*M*_w_ (10^5^ Da)	*M*_n_ (10^5^ Da)	*Ð* (−)
1-Propanol	1.72	1.01	1.7	1.81	1.09	1.7
2-Propanol	1.64	0.92	1.8	1.76	0.97	1.8
Ethanol	1.39	0.67	2.1	1.34	0.62	2.2
*n*-Heptane	1.58	0.85	1.9	1.99	1.17	1.7

*Four different precipitants were tested respectively. Extractions were performed under reflux conditions.*

### Extraction With Impure, Non-halogenated Solvents

**FIGURE 3:**
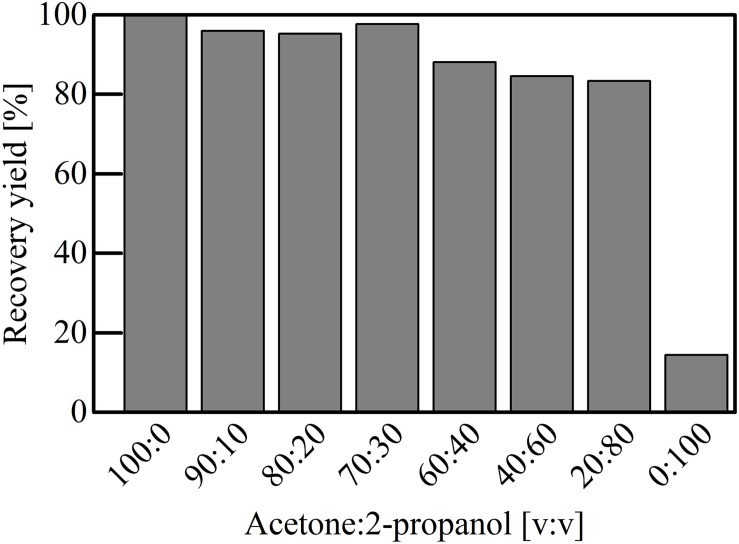
Extraction of P(HB-*co*-HHx) with a mixture of acetone and 2-propanol. The P(HB-*co*-HHx) recovered with pure acetone was set as the reference extraction. Different ratios of 2-propanol supplementation imitate impure acetone after solvent recovery via rotary evaporation.

For the economic and ecological footprint of the downstream process, it is important that the utilized solvents can be recycled. Distillation and evaporations are common methods for solvent recovery. At the end of the PHA downstream process, a mixture of solvent and precipitant are present. During solvent recovery, solvents with fractions of the precipitant or vice versa might be obtained. To simulate this scenario, solvent mixtures of acetone with volume fractions of 2-propanol from 0 to 100% were prepared and P(HB-*co*-HHx) was extracted with these mixtures from freeze dried cells (Figure 3). The recovery with acetone/2-propanol was as efficient as with pure acetone (>95%) with volume fractions of 2-propanol up to 30%. With increased impurities, the extraction efficiency decreased, but it was still possible to extract 83% of the PHA (compared to the extraction with pure acetone) with a 2-propanol volume fraction of 80%. Even with pure 2-propanol it was still possible to extract 14.5% the amount of PHA compared to pure acetone.

### PHA Recovery With Non-halogenated Solvents at Different Temperatures

In order to intensify the evaluation for the PHA extraction process with acetone, the influences of the extraction times and extraction temperature were investigated. From the recovered P(HB-*co*-HHx), the MWCs and polymer purity as well as monomer composition was examined.

#### Molecular Weight Characteristics

**FIGURE 4:**
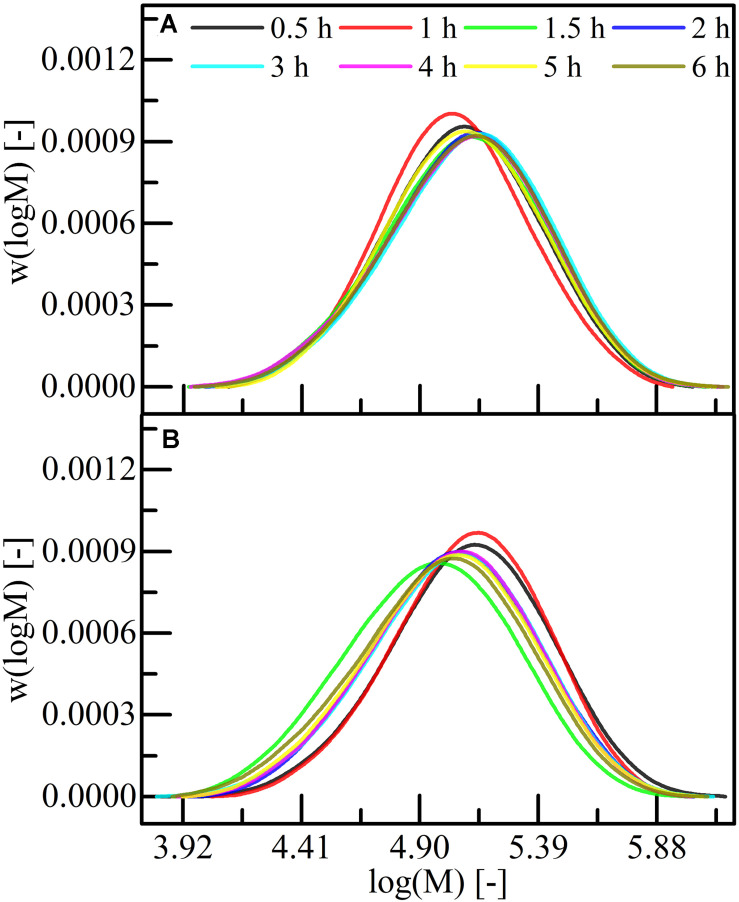
Distribution of the logarithmic molecular weight for P(HB-*co*-HHx) extracted with acetone at different temperatures. All extractions were performed under reflux conditions either at **(A)** RT or at **(B)** 70°C. Samples were taken at different time points as indicated in graphs. 2-propanol was used as precipitant.

In general, a similar MWD was obtained for all sampling time points, except for the 1 h sampling time point, when the polymer was extracted at RT (Figure 4). In contrast, the short extraction times (0.5 and 1 h) at 70°C yielded in a higher MW, compared to longer extraction time (Figure 4 and Table 5). The extraction at RT facilitated recovery of PHA of a higher MW (*M*_w,max_ = 1.63 ± 0.01 × 10^5^ Da, *M*_n,max_ = 0.91 ± 0.00 × 10^5^ Da) compared to the 70°C extraction (*M*_w,max_ = 1.55 ± 0.14 × 10^5^ Da, *M*_n,max_ = 0.85 ± 0.11 × 10^5^ Da). Nonetheless, both set-ups overall yielded in narrow MWD (*Ð* = 1.7–1.9). The lowest MW polymer was extracted at 70°C (*M*_w,min_ = 1.27 ± 0.10 × 10^5^ Da, *M*_n,m__in_ = 0.67 ± 0.03 × 10^5^ Da), which was significantly lower compared to the lowest MW polymer extracted at RT (*M*_w,min_ = 1.40 ± 0.03 × 10^5^ Da, *M*_n,min_ = 0.83 ± 0.02 × 10^5^ Da). Short extraction times were sufficient for the recovery of high MW polymers and the extraction at RT is preferable for the extraction of higher MW polymer.

**TABLE 5:** Molecular weight characteristics for P(HB-*co*-HHx) extracted with acetone at different temperatures and precipitated with 2-propanol.

Time Point (h)	Molecular weight characteristics					
	21°C (RT)			70°C		
	*M*_w_ (10^5^ Da)	*M*_n_ (10^5^ Da)	*Ð* (−)	*M*_w_ (10^5^ Da)	*M*_n_ (10^5^ Da)	*Ð* (−)
0.5	1.56 ± 0.01	0.91 ± 0.00	1.7 ± 0.0	1.55 ± 0.14	0.85 ± 0.11	1.8 ± 0.1
1	1.40 ± 0.03	0.83 ± 0.02	1.7 ± 0.1	1.52 ± 0.10	0.87 ± 0.10	1.8 ± 0.1
1.5	1.59 ± 0.00	0.89 ± 0.01	1.8 ± 0.0	1.27 ± 0.10	0.67 ± 0.03	1.9 ± 0.1
2	1.60 ± 0.02	0.91 ± 0.03	1.8 ± 0.0	1.47 ± 0.02	0.82 ± 0.01	1.8 ± 0.0
3	1.62 ± 0.04	0.93 ± 0.03	1.7 ± 0.0	1.42 ± 0.04	0.76 ± 0.02	1.9 ± 0.0
4	1.60 ± 0.07	0.89 ± 0.00	1.8 ± 0.0	1.40 ± 0.02	0.75 ± 0.04	1.9 ± 0.1
5	1.59 ± 0.00	0.92 ± 0.01	1.7 ± 0.0	1.44 ± 0.03	0.78 ± 0.02	1.8 ± 0.0
6	1.63 ± 0.01	0.92 ± 0.00	1.8 ± 0.0	1.35 ± 0.01	0.70 ± 0.00	1.9 ± 0.0

*All extractions were performed under reflux conditions either at RT or at 70°C. Samples were taken at different time points as indicated in the table. Errors indicate maximum and minimum values of duplicate experiments.*

#### PHA Purity and Composition

**FIGURE 5:**
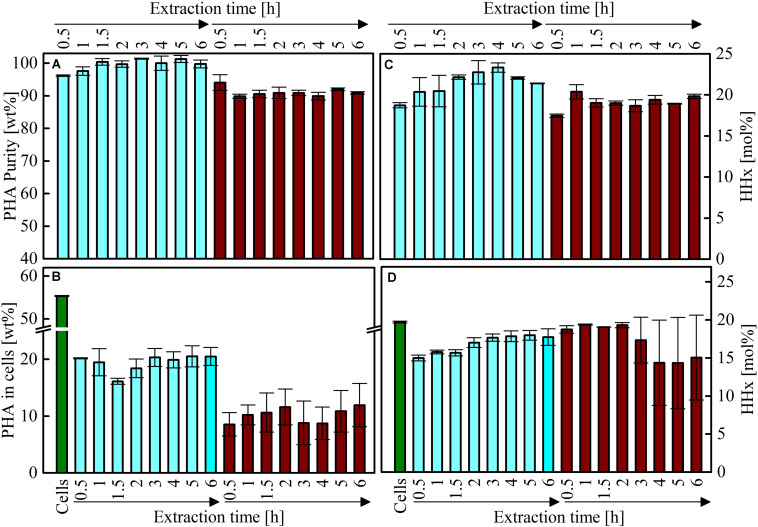
P(HB-*co*-HHx) contents, purities and molecular compositions of material extracted with acetone at different temperatures and precipitated with 2-propanol. All extractions were performed under reflux conditions either at RT (light blue bars) or at 70°C (dark red bars). The initial PHA content from the cells before extraction is shown in the green bars. Samples were taken at different time points as indicated in the figure. **(A)** PHA purities of the extracted material. **(B)** Remaining PHA contents in the cells after extraction. **(C)** Molar HHx contents of the extracted material. **(D)** Molar HHx contents of the not-extracted PHA. Errors indicate maximum and minimum values of duplicate experiments.

In order to evaluate recovered polymers, the temperature and time dependent influence on the polymer purity and composition was analyzed. It was observed, that the polymer purity was higher in the material extracted at RT (up to 100%) compared to the material recovered at 70°C (up to 94%) (Figure 5A). The analysis also revealed, that the remaining PHA in the cells was in average higher with the cold extraction (19.4 ± 1.4%) compared to the 70°C extraction (10.2 ± 1.2%) (Figure 5B). No significant differences were observed between the two extraction temperatures according to the molecular composition (Figure 5C). Nevertheless, the average molar HHx fraction of the RT recoveries was higher (21.4 ± 1.4 mol%) compared to the 70°C extraction (19.1 ± 0.8 mol%). The overall average molar HHx fraction of the recovered PHA was 20.3 ± 1.6 mol%, which is above the average HHx content in remaining cells (17.0 ± 1.7 mol%) (Figures 5C,D). Consequently, a one-step extraction with acetone at RT is favorable for recovery of high purity P(HB-*co*-HHx), but recovery yields are lower compared to extractions at 70°C, where the recovered P(HB-*co*-HHx) is of lower product quality but the recovery yield in a one-step extraction is improved. Overall, no trend with longer extraction times was observed.

### Multi-Stage PHA Extractions With Non-halogenated Solvents

It was shown in the previous section, that 10–20% PHA remained in the cells after a one-step extraction. Therefore, the extraction with ethyl acetate and acetone was evaluated in a three-stage extraction process in order to recover the whole PHA form the cells. Both solvents resulted in recovery of the largest fraction (83–86%) of PHA in the first extraction step (Table 6). An additional 12–16% PHA were extracted in the second extraction stage and only a very small fraction (1–2%) was extracted in the third stage. Thus, a two-step extraction process allows the extraction of up to 99% P(HB-*co*-HHx) with non-halogenated solvents from freeze dried cells.

**TABLE 6:** Recovered P(HB-*co*-HHx) in a multi-stage extraction with different solvents.

	Recovery Yield (%)	
Extraction stage	Ethyl acetate	Acetone
1	85.7 ± 0.3	82.8
2	12.3 ± 0.3	16.1
3	2	1.1
Sum	100	100

*Cells were extracted with ethyl acetate or acetone and precipitated with n-heptane or 2-propanol, respectively. Ethyl acetate experiments were performed in triplicates and ± SD are indicated.*

### NMR of Recovered P(HB-*co*-HHx)

**FIGURE 6:**
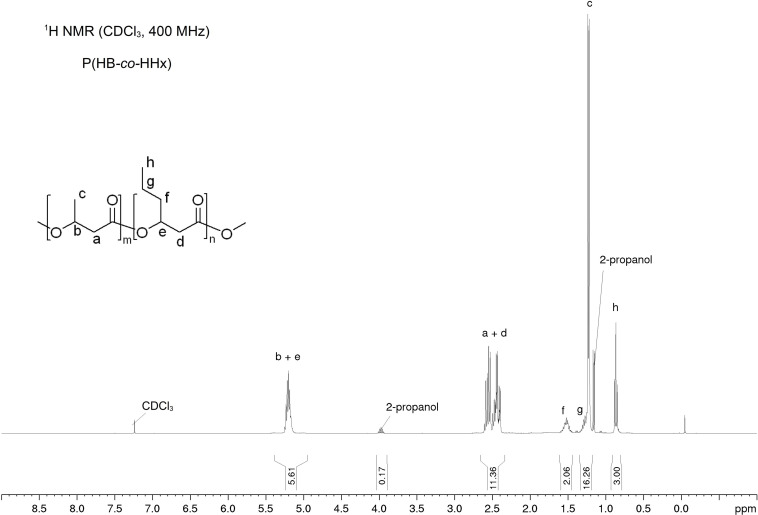
^1^H NMR spectrum of P(HB-*co*-HHx) extracted with acetone at RT for 2 h and precipitated with 2-propanol. ^1^H NMR (CDCl_3_, 400 MHz): δ = 5.17–5.23 (m, 1H), 5.17–5.23 (m, 1H), 2.46–2.61 (m, 2H), 2.39–2.45 (m, 2H), δ = 1.48–1.56 (m, 2H), 1.27–1.31 (m, 2H), 1.23 (d, *J* = 6.3 Hz, 3H), 0.87 ppm (t, *J* = 7.3 Hz, 3H).

**FIGURE 7:**
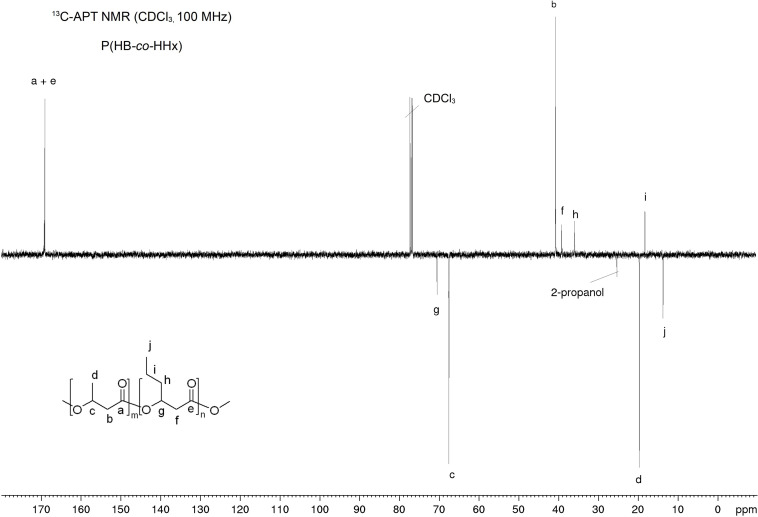
^13^C-APT NMR spectrum of P(HB-*co*-HHx) extracted with acetone at RT for 2 h and precipitated with 2-propanol. ^13^C-APT NMR (CDCl_3_, 100 MHz): δ = 169.2 (+), 169.1 (+), 70.5 (−), 67.6 (−), 40.7 (+), 39.2 (+), 36.0 (+), 19.7 (−), 18.3 (+), 13.7 (−) ppm.

The NMR analysis with material extracted and precipitated with either chloroform/*n*-heptane, ethyl acetate/2-propanol, or acetone/2-propanol confirmed the presence of the 3-HB and 3-HHx building blocks. As an example, the ^1^H and ^13^C-APT NMR spectra of acetone/2-propanol extracted material are shown in Figures 6, 7. The other NMR spectra and zooms of the respective ^13^C-APT NMR spectra for the calculation of the randomness factor *D* can be found in the Supplementary Figures S1–S7. The peaks are labeled according to the presence of the ^1^H protons in the respective monomers (Figure 6). Additionally, peaks of 2-propanol at δ = 1.16 ppm and δ = 3.95 ppm were identified. An HHx content of 17.3–19.2 mol% was determined from ^1^H NMR spectra (Table 5). All carbon atoms were identified with the ^13^C-APT NMR analysis (Figure 7) and the results were used to identify the randomness factor *D* of the extracted copolymer (Table 7). The analysis of material extracted and precipitated with different solvents showed a *D* of 0.78–2.29, which corresponds to a random copolymer structure.

**TABLE 7:** P(HB-*co*-HHx) characteristics determined from ^1^H and ^13^C-APT NMR spectra.

	P(HB-*co*-HHx) properties	
Solvent/precipitant	*D* (−)	HHx (mol%)
Acetone/2-propanol	2.29	17.3
Chloroform/*n*-heptane	0.78	19.2
Ethyl acetate/2-propanol	1.23	18.3

## Discussion

The recovery of PHAs accounts for a large portion of the process economics and overall ecological footprint (Pérez-Rivero et al., 2019). In addition, the PHA productivity, intracellular content, and yield from the substrate, as well as the carbon substrate price all contribute to the final production price (Choi and Lee, 1999). High purity PHA is traditionally recovered with halogenated solvents, which are expensive and have a negative environmental impact. These extraction processes were developed based on the poor solubility of *scl*-PHAs in most solvents. In contrast, *mcl*-PHAs and copolymers thereof exhibit an increased solubility in *scl*-PHA non-solvents (Jacquel et al., 2007). In this study, the P(HB-*co*-HHx) extraction with acetone as a non-halogenated and low toxic solvent was developed with regards to the MWCs, PHA purity, and PHA yield by investigating the extraction time, temperature, and steps, as well as the suitability of different precipitant.

The shown non-halogenated solvents (Table 1) have all a lower density than water (ρ = 1 g cm^–3^), which facilitates a more simple recovery process (Riedel et al., 2013). Acetone in combination with 2-propanol was chosen as the most suitable solvent-precipitant pair: the mixture of the solvents is zeotrope and a Δ*T*_B_ of 27°C (Table 2) facilitates solvent separation and recovery by distillation. In contrast to 1-propanol, 2-propanol is less toxic (Strubelt et al., 1999) and the lower *T*_B_ requires less energy input for distillation. During P(HB-*co*-HHx) recovery from a 20 m^3^ cultivation, Chen and co-workers calculated the downstream costs are associated with more than 50% of the total process costs if solvents are not recycled (Chen et al., 2001). Such a recycling has a huge impact on lowering the downstream costs and reduces the environmental impact by a factor of 100 compared to the respective solvent production (Chen et al., 2001; Wampfler et al., 2010). Additionally, the usage of acetone or ethyl acetate facilitates the recovery of low endotoxin containing *mcl*-PHA, which subsequently allows its potential use in medical applications (Furrer et al., 2007). Another advantage of acetone as a solvent is, that it can also be used for the extraction of *scl-*PHAs by elevating the pressure or temperature in the system (Koller et al., 2013a; Aramvash et al., 2016). In addition, it was shown that acetone containing a large fraction of 2-propanol can still efficiently recover the polymer as good as pure acetone and even pure 2-propanol recovered a little amount of polymer (Figure 4). Pure 2-propanol could also recover 23% poly(3-hydroxyoctanoate) (PHO) from *Pseudomonas* sp. (Furrer et al., 2007). The negligibility of 2-propanol impurities could also be valuable characteristic to reduce the solvent recycling costs. Consequently, acetone in combination with 2-propanol has excellent properties for P(HB-*co*-HHx) recovery and should be used for an economic and ecologically favorable downstream process.

Ethanol was not chosen as a precipitant due to a lower *M*_w_ and *M*_n_ of the recovered polymer and a broader MWD (Table 4). Elbahloul and Steinbüchel (2009) extracted high purity PHO from 40 kg biomass (*Pseudomonas putida*) with acetone and subsequent precipitation with a mixture of 70% methanol and 70% ethanol, but MWCs were not provided. Cespedes and co-workers also used acetone for the recovery of P(HB-*co*-HA_*mcl*_) copolymer from *Pseudomonas* sp. and facilitated precipitation by ethanol. They obtained a 15% lower MW when the polymer was purified with acetone/ethanol compared to the recovery with chloroform/ethanol, but did not observe a broader MWD (Cespedes et al., 2018). Jiang and co-workers also used acetone to recover *mcl*-PHA from *P. putida* biomass and precipitated the polymer with methanol. By this solvent-precipitant combination, a *Ð* of 1.8–1.9 was obtained (Jiang et al., 2006), which is in accordance with the results for the precipitation with 1-propanol, 2-propanol, and heptane in this study (Table 4).

NMR analysis confirmed an HHx content of 17.3–19.2 mol% for differently recovered PHA, all comprising random copolymer structure with *D* values between 0.78 and 2.29. Kamiya et al. (1989) described random copolymer structure for the copolymer poly(hydroxybutyrate-*co*-hydroxyvalerate) for *D* values between 0.67 to 1.5 and mixtures of random copolymers with *D* values greater than 1.5. Block polymers were found to have a *D* value much larger than 1. The HHx values obtained by NMR analysis and also by GC analysis are within the typically range reported for the strain (Riedel et al., 2012, 2015; Murugan et al., 2016; Thinagaran and Sudesh, 2017; Purama et al., 2018). The extraction with acetone generally resulted in P(HB-*co*-HHx) with a larger fraction of HHx than the remaining PHA in the cells (Figure 5), which could be explained by the affinity of acetone to polymer chains with a higher *mcl*-fraction.

Influences on the MW during acetone extraction at RT were not detected for the investigated extraction period of 6 h, but a decrease of the MW for extraction time >1 h at 70°C were detected (Table 5). In contrast, a reduction of the MW was not observed for chloroform extractions at RT and subsequent treatment at 100°C for 4 h (Figure 1). This thermal stability of the extracted PHA was unexpected, as Ramsay and co-workers could show that the *M*_W_ of PHB (1.2 × 10^6^ Da) extracted from *R. eutropha* drastically (12–50%) decreased for extraction times of 0.25–96 h in halogenated solvents at their boiling point (Ramsay et al., 1994). Penloglou et al. (2012) could also see a 5% reduction of the *M*_w_ of PHB extracted with chloroform for 24 h at 30°C from *Azohydromonas lata*, but they showed that pretreating the biomass with sonication for 60 min yields in a significantly larger *M*_w_ reduction of 40%. The temperature effects on the longer polymer chains might be more critically than on the shorter chains, which were investigated in this study. Thus, extraction with acetone at RT should be preferred with the only negative aspect of lower extraction yields compared to the 70°C extraction (Figure 5). For further process developments, it is important to consider the yield variations, when the extractions are performed with wet biomass as reported previously: The yield decreased from 95 to 71% when P(HB-*co*-HHx) was extracted from wet biomass (Riedel et al., 2013). Nevertheless, an implementation of a two-stage extraction with short extraction times could significantly improve the overall recovery yield (Table 6) and should therefore be implemented for acetone extractions.

## Conclusion

In this study it was shown, that acetone as a *scl*-PHA non-solvent can efficiently extract P(HB-*co*-HHx) from freeze dried biomass. An extraction system in combination with 2-propanol for PHA precipitation was developed with regard to select zeotropic solvents with low toxicity. Therefore, solvent recycling of the solvents is feasible and allows drastic reduction of the downstream costs. A two-stage extraction process is proposed for the extraction of most of the PHA from the cells. The developed strategy could help to decrease PHA production costs and contribute to a more ecological process, which both would support the commercialization process of PHAs.

## Data Availability Statement

The raw data supporting the conclusions of this article will be made available by the authors, without undue reservation.

## Author Contributions

SR, TG, and MB contributed to the conception and design of the study. MB, TW, and BG carried out the experiments and analysis of the data. BG and MB prepared the first draft of the manuscript. SR, TG, and PN were responsible for the project administration and funding acquisition. All authors contributed to the manuscript revision, read and approved the submitted version.

## Conflict of Interest

MB, TW, and TG were employed by the company ANiMOX GmbH, Berlin, Germany. The remaining authors declare that the research was conducted in the absence of any commercial or financial relationships that could be construed as a potential conflict of interest.

## Footnotes

1 https://danimerscientific.com

2 https://www.kaneka.co.jp
