# Supplementary material for: Recovery of the PHA Copolymer P(HB-co-HHx) With Non-halogenated Solvents: Influences on Molecular Weight and HHx-Content
Source: Front Bioeng Biotechnol. 2020 Aug 7;8:944. doi: 10.3389/fbioe.2020.00944 (PMC7438878; doi:10.3389/fbioe.2020.00944)
Supplement: Supplementary file 1 [file Data_Sheet_1.pdf]

frontiers

Supplementary Material

Recovery of the PHA Copolymer P(HB-*co*-HHx) with Non-Halogenated Solvents: Influences on Molecular Weight and HHx-Content

Moritz Bartels[†,1], Björn Gutschmann[†,2], Torsten Widmer[1], Thomas Grimm[1], Peter Neubauer[2], Sebastian L. Riedel[2*]
[1]ANiMOX GmbH, Max-Plank-Straße 3, 12489 Berlin, Germany
[2]Technische Universität Berlin, Institute of Biotechnology, Chair of Bioprocess Engineering, Ackerstraße 76, 13355 Berlin, Germany
*** Correspondence:** Sebastian L. Riedel, riedel@tu-berlin.de

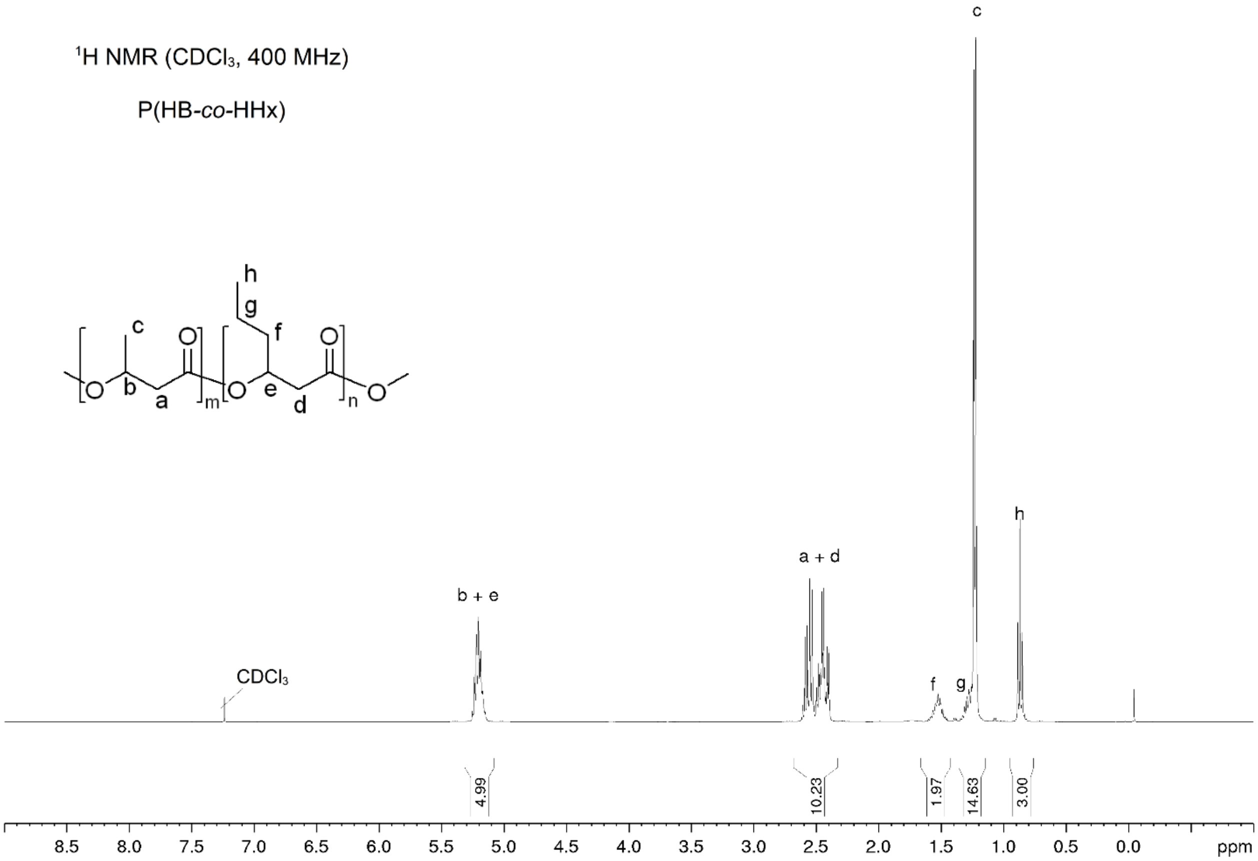

Figure S1 | ^{1}H NMR spectrum of P(HB-*co*-HHx) with chloroform at RT for 2 h and precipitated with heptane. ^{1}H NMR ($CDCl_3$, 400 MHz): δ = 5.17 – 5.23 (m, 1H), 5.17 – 5.23 (m, 1H), 2.46 – 2.61 (m, 2H), 2.39 – 2.45 (m, 2H), 1.48 – 1.56 (m, 2H), 1.27 – 1.31 (m, 2H), 1.23 (d, *J*=6.3 Hz, 3H), 0.87 ppm (t, *J* = 7.3 Hz, 3H).

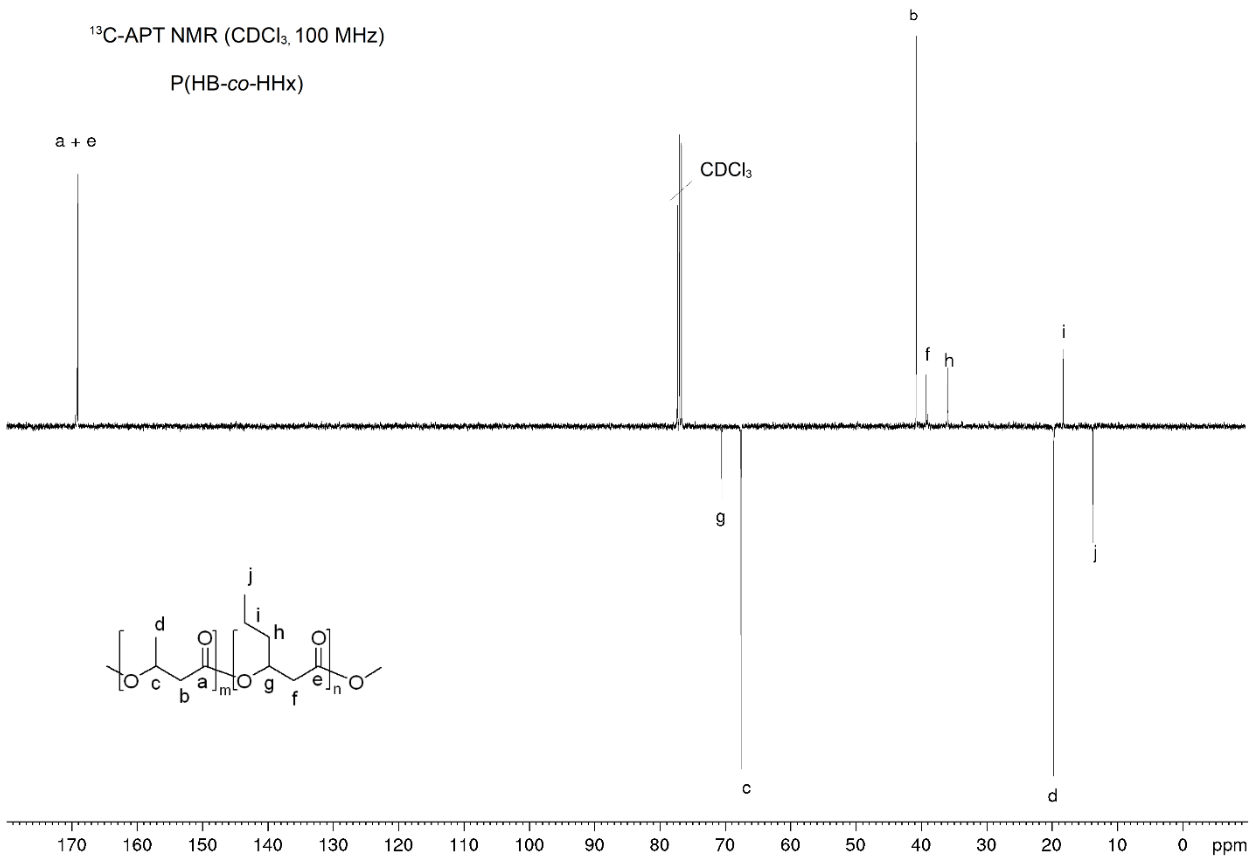

Figure S2 | ^{13}C-APT NMR spectrum of P(HB-*co*-HHx) extracted with chloroform at RT for 2 h and precipitated with heptane. ^{13}C-APT NMR ($CDCl_3$, 100 MHz): δ = 169.2 (+), 169.1 (+), 70.5 (-), 67.6 (-), 40.7 (+), 39.2 (+), 36.0 (+), 19.7 (-), 18.3 (+), 13.7 (-) ppm.

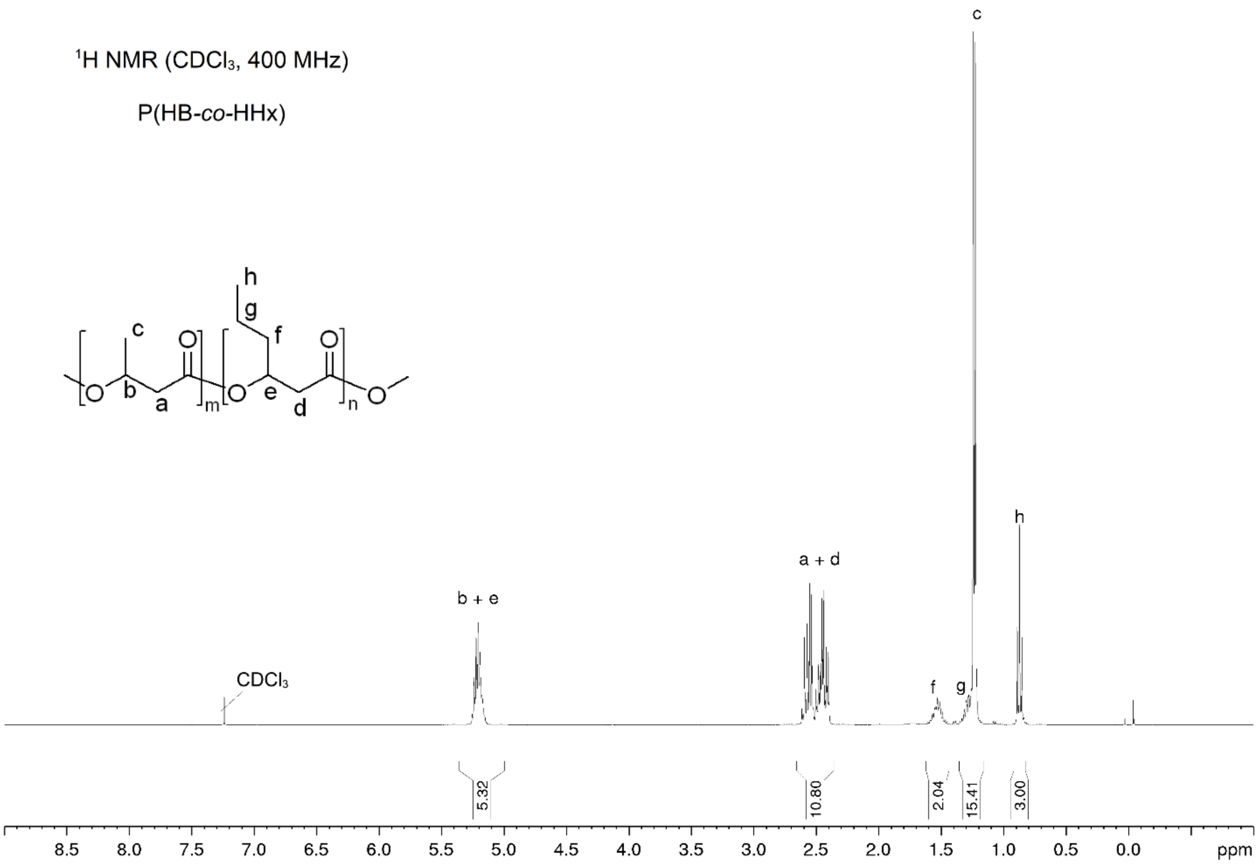

Figure S3 | ^{1}H NMR spectrum of P(HB-*co*-HHx) extracted with ethyl acetate at RT for 2 h and precipitated with 2-propanol. ^{1}H NMR ($CDCl_3$, 400 MHz): δ = 5.17 – 5.23 (m, 1H), 5.17 – 5.23 (m, 1H), 2.46 – 2.61 (m, 2H), 2.39 – 2.45 (m, 2H), 1.48 – 1.56 (m, 2H), 1.27 – 1.31 (m, 2H), 1.23 (d, *J*=6.3 Hz, 3H), 0.87 ppm (t, *J* = 7.3 Hz, 3H).

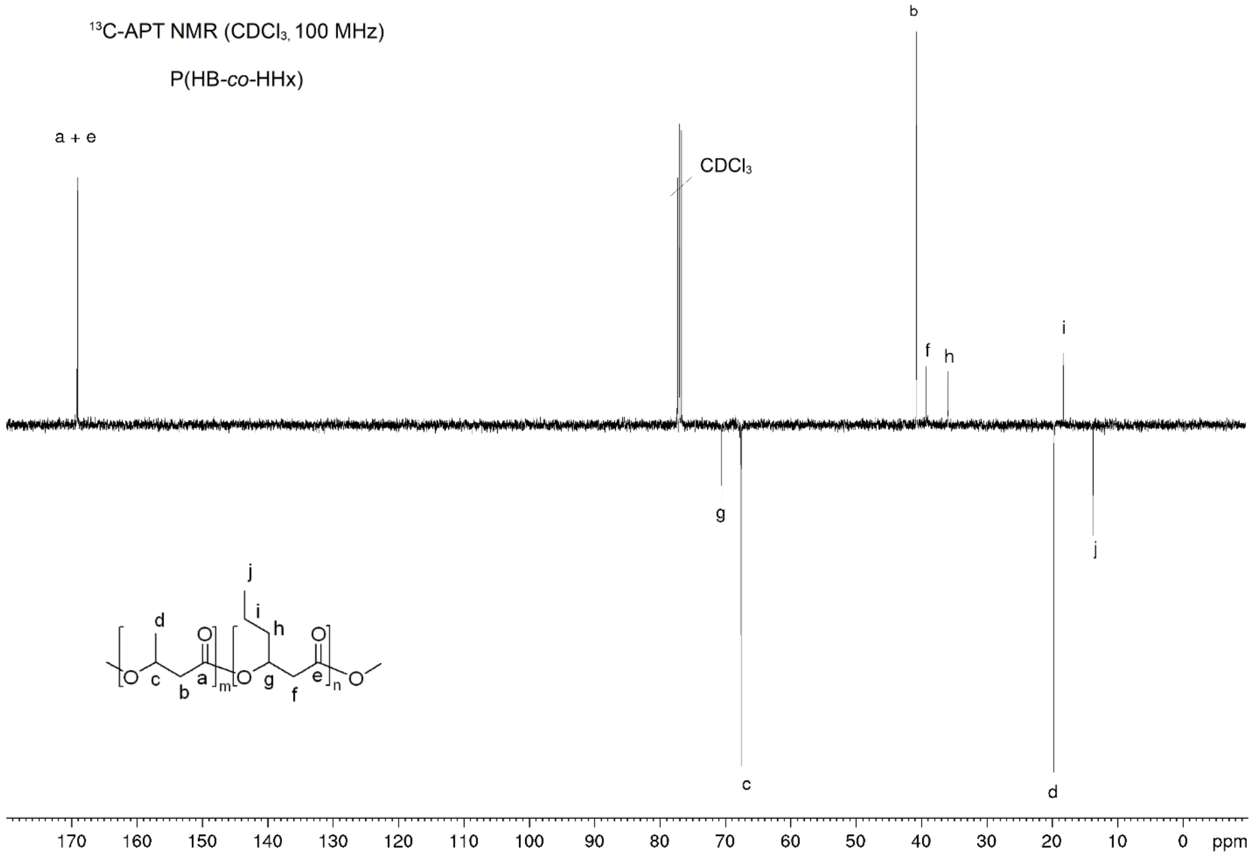

Figure S4 | ^{13}C-APT NMR spectrum of P(HB-*co*-HHx) extracted with ethyl acetate at RT for 2 h and precipitated with 2-propanol. ^{13}C-APT NMR ($CDCl_3$, 100 MHz): δ = 169.2 (+), 169.1 (+), 70.5 (-), 67.6 (-), 40.7 (+), 39.2 (+), 36.0 (+), 19.7 (-), 18.3 (+), 13.7 (-) ppm.

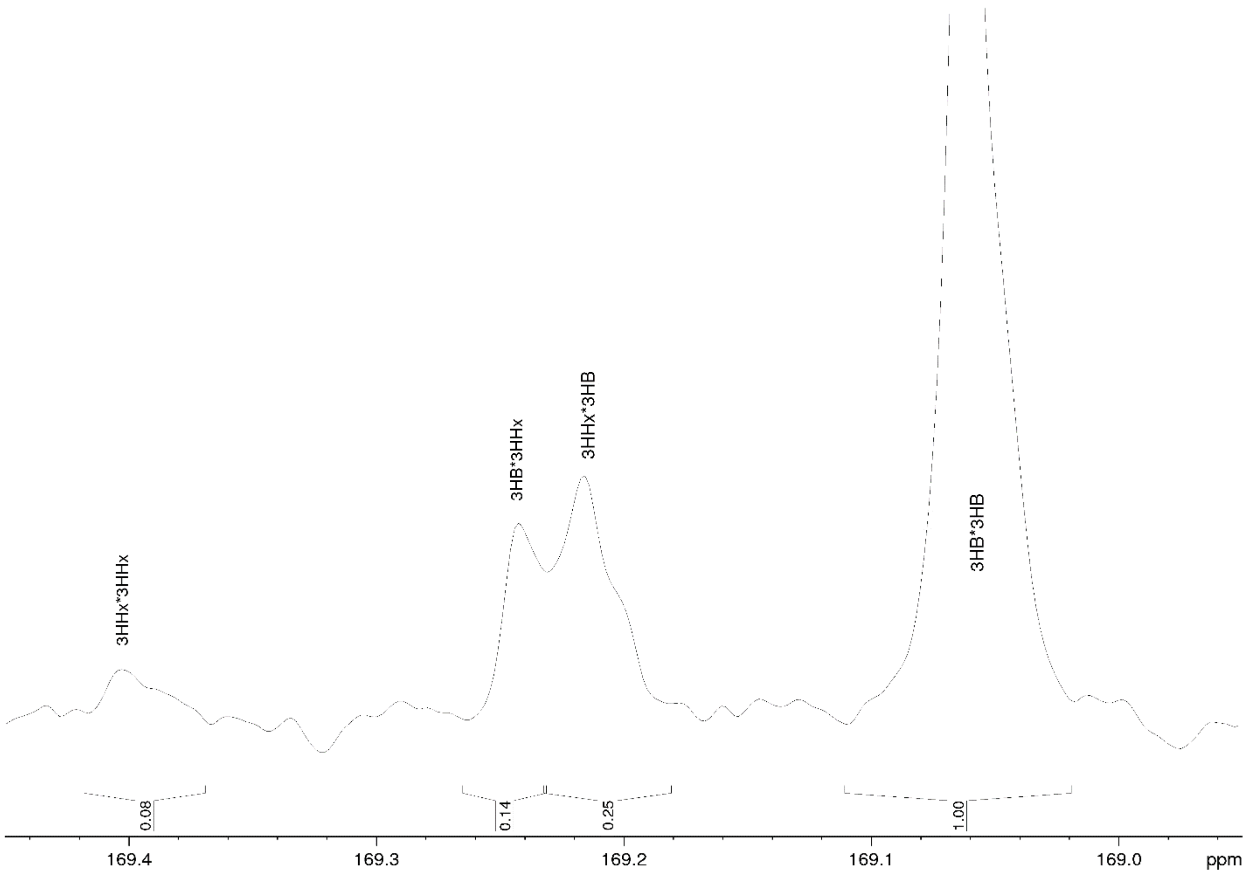

Figure S5 | Zoom of ^{13}C-APT NMR spectrum of P(HB-*co*-HHx) extracted with acetone at RT for 2 h and precipitated with 2-propanol. Peaks were used for calculation of randomness.

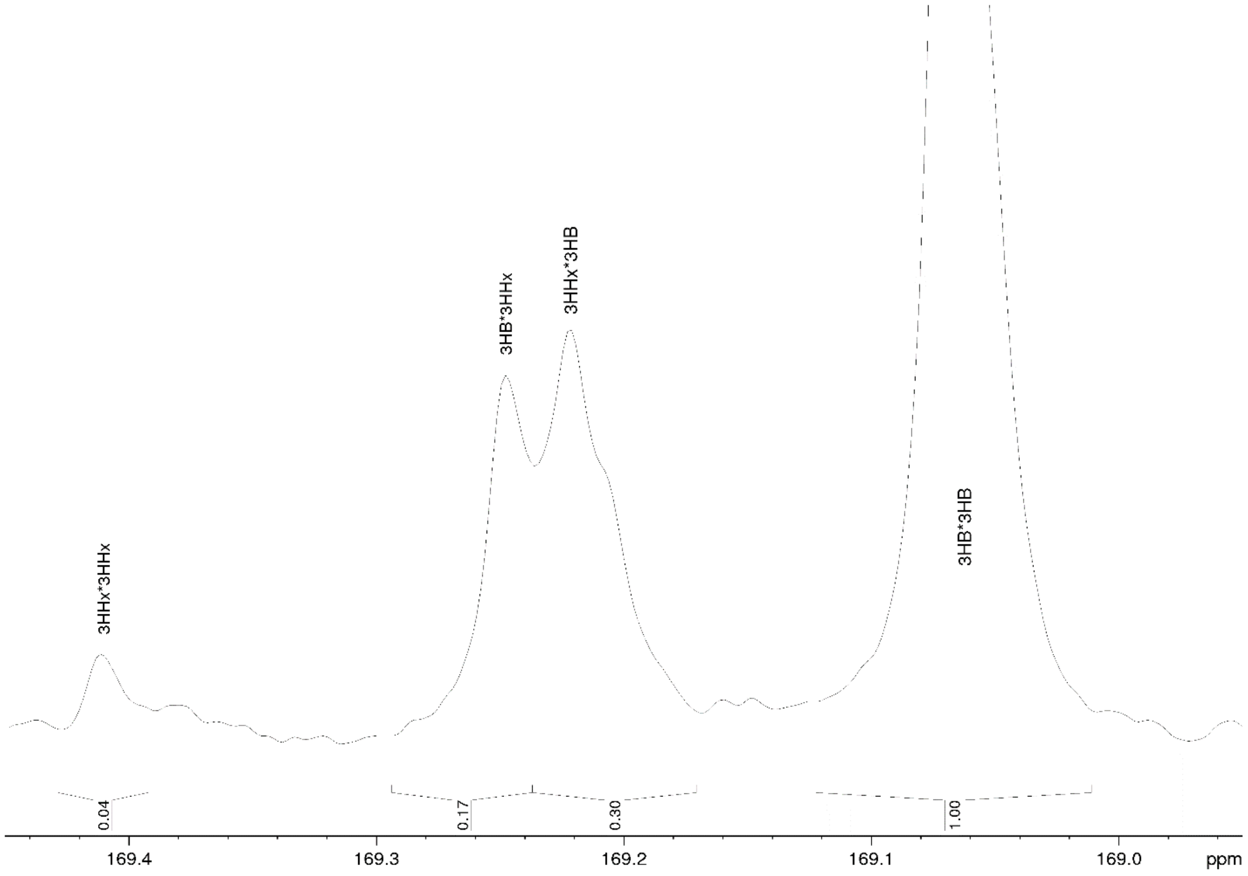

Figure S6 | Zoom of ^{13}C-APT NMR spectrum of P(HB-*co*-HHx) extracted with chloroform at RT for 2 h and precipitated with heptane. Peaks were used for calculation of randomness.

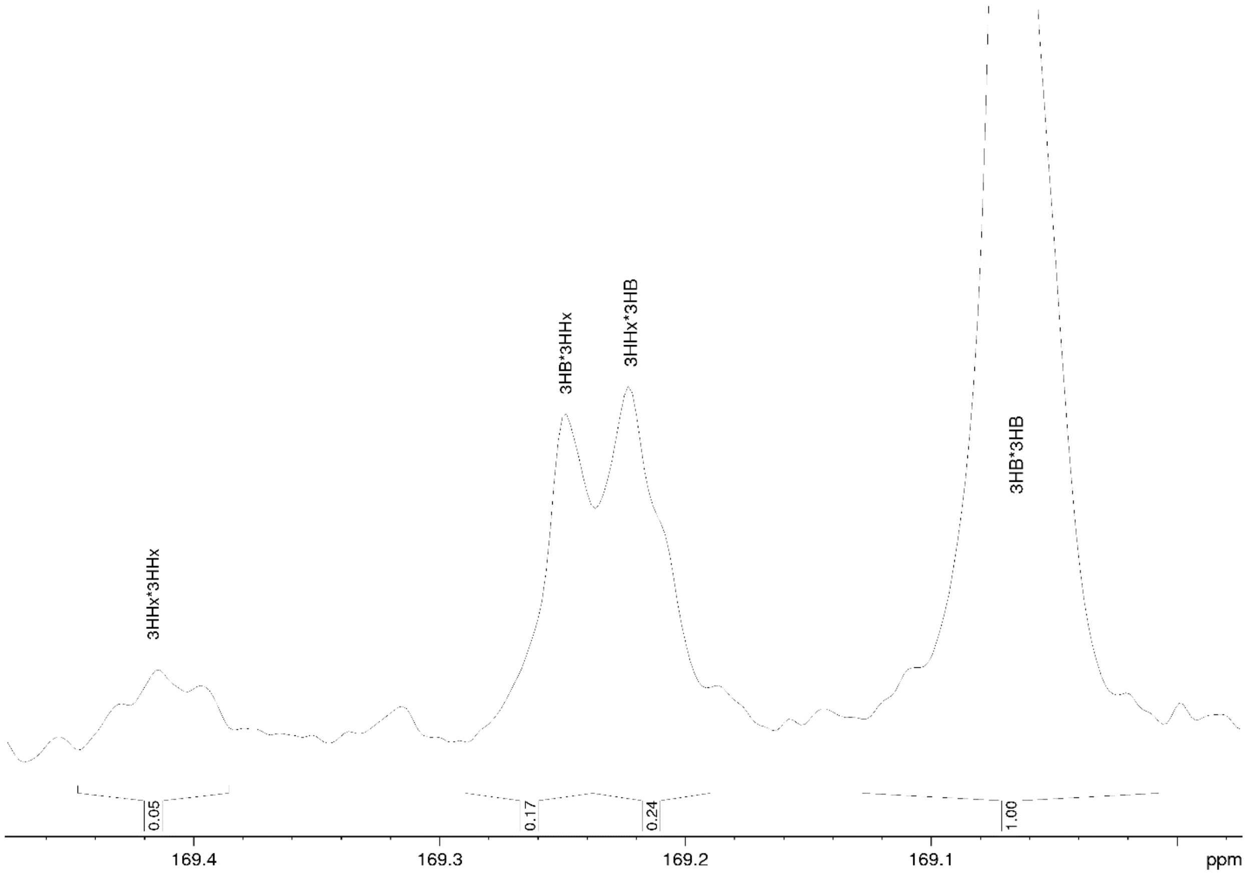

Figure S7 | Zoom of ^{13}C-APT NMR spectrum of P(HB-*co*-HHx) extracted with ethyl acetate at RT for 2 h and precipitated with 2-propanol. Peaks were used for calculation of randomness.
